# Feasibility and efficacy of fecal microbiota transplantation in adolescents with refractory irritable bowel syndrome: A randomized clinical pilot trial

**DOI:** 10.1002/jpn3.70459

**Published:** 2026-06-01

**Authors:** Clara M. A. de Bruijn, Anna O. J. Oorthuys, Judith Zeevenhooven, Mark Davids, Johannes H. M. Levels, Arine M. Vlieger, Hilde Herrema, Max Nieuwdorp, Marc A. Benninga

**Affiliations:** ^1^ Emma Children's Hospital, Amsterdam University Medical Centers University of Amsterdam, Pediatric Gastroenterology, Hepatology and Nutrition Amsterdam the Netherlands; ^2^ Amsterdam Reproduction & Development Research Institute, Amsterdam University Medical Centers Location Academic Medical Center/Emma Children's Hospital Amsterdam the Netherlands; ^3^ Amsterdam Gastroenterology Endocrinology Metabolism Research Institute, Amsterdam University Medical Centers University of Amsterdam, Gastroenterology and Hepatology Amsterdam the Netherlands; ^4^ Departments of Internal and Experimental Vascular Medicine Amsterdam University Medical Centers, Location Academic Medical Center Amsterdam the Netherlands; ^5^ Department of Pediatrics St. Antonius Hospital Nieuwegein the Netherlands

**Keywords:** abdominal pain, gut microbiome, microbiota‐based therapy, pediatric gastroenterology

## Abstract

**Objectives:**

To assess the feasibility and efficacy of fecal microbiota transplantation (FMT) in adolescents (16–21 years) with refractory irritable bowel syndrome (IBS).

**Methods:**

Randomized controlled pilot trial. Thirty‐two patients were included and randomized to receive two allogeneic or autologous FMTs. At baseline and after 6 weeks, two allogeneic or autologous FMTs were administered via a nasoduodenal tube. Feasibility outcomes included dropout rate. Clinical efficacy was evaluated by the proportion of responders (≥50 points reduction in total score of the IBS severity‐scoring‐system) at 12, 24, and 48 weeks follow‐up. Secondary outcomes included health‐related quality of life (QoL), depression and anxiety scores, and school/work absenteeism.

**Results:**

One patient (3%) withdrew after randomization, due to lack of effect after the first FMT. Response rates 12 weeks after allogeneic and autologous FMTs were 40% and 38% (*p* = 0.886). At 24 weeks, significantly more patients responded after allogeneic FMTs (60% vs. 25% autologous, *p* = 0.048), without significant differences at 48 weeks (60% vs. 50%, *p* = 0.576). Total QoL score was significantly better after allogeneic than autologous FMTs at 12, 24, and 48 weeks (*p* = 0.028, *p* = 0.007, *p* = 0.011). In the allogeneic FMTs group, school/work absenteeism was 7% at 24 weeks (vs. 41% autologous, *p* = 0.037).

**Conclusions:**

Allogeneic FMTs were feasible and resulted in high response rates and better QoL compared to autologous FMTs. These results provide preliminary evidence for the use of allogeneic FMTs in adolescents with refractory IBS.

**Trial Registration:**

ClinicalTrials.gov identifier: NCT03074227 (https://clinicaltrials.gov/study/NCT03074227?id=NCT03074227&rank=1).

## Abstract (highlights)

**What is Known:**

- Irritable bowel syndrome (IBS) is a common chronic condition in adolescents, often refractory to standard treatments.
- Fecal microbiota transplantation (FMT) has shown efficacy in improving symptoms in adults with IBS.
- Data on the feasibility and efficacy of FMT in adolescents with IBS are lacking.

**What is New:**

- This is the first randomized, double‐blind, placebo‐controlled pilot trial of FMT in adolescents with refractory IBS.
- Allogeneic FMT was feasible, with high patient acceptance and low dropout rates.
- Allogeneic FMT led to significant and sustained improvements in abdominal pain, QoL, and school attendance compared to autologous FMT.

## 1 INTRODUCTION

Irritable bowel syndrome (IBS), defined by the Rome IV criteria, is a chronic abdominal pain disorder in children and adolescents, currently recognized as a disorder of gut–brain interaction.^1^ IBS can be classified into four subtypes: IBS‐constipation, IBS‐diarrhea, IBS‐mixed, and IBS‐unclassified.^2^, ^3^ Worldwide pooled prevalence rates of IBS range between 6%−12% in children and 6%−17.5% in adults, with peak prevalence in adolescents.^4^, ^5^ IBS significantly reduces quality of life (QoL), is associated with increased school and/or work absence, and a higher risk of depressive or anxiety disorders compared to healthy subjects, resulting in a substantial socioeconomic burden.^6^, ^7^, ^8^, ^9^

Standard treatment includes education, reassurance, lifestyle interventions, and dietary advice.^10^ Although evidence in pediatrics is limited, the low FODMAP (Fermentable Oligosaccharides, Disaccharides, Monosaccharides, and Polyols) diet and probiotics (i.e., *Lacticaseibacillus rhamnosus GG*, *Limosilactobacillus reuteri*) are sometimes used.^10^, ^11^, ^12^ Non‐pharmacological (hypnotherapy, cognitive behavioral therapy) and pharmacological (antispasmodics, antidepressants) treatments are successfully used, but symptoms may persist in some children.^11^, ^13^, ^14^, ^15^ For these refractory patients, alternative treatments are needed.

The etiology of IBS is multifactorial, involving genetic, physiological, psychological, and socio‐environmental factors, leading to disturbances in the central nervous system and gastrointestinal (GI) tract.^16^ Dysregulation of the gut–brain axis may cause visceral hypersensitivity and abnormal GI motility.^16^ Growing evidence suggests a role for the intestinal microbiota in IBS, as approximately 10% of patients develop post‐infectious IBS.^17^, ^18^, ^19^, ^20^, ^21^, ^22^ Adult IBS patients show intestinal dysbiosis, with decreased diversity and altered composition.^18^ Pediatric studies also reveal specific microbiome signatures.^17^, ^20^, ^21^

Manipulation of the intestinal microbiota by donor fecal microbiota transplantation (FMT), may restore dysbiosis and improve IBS symptoms. Eight randomized controlled trials (RCTs) in adults show that FMT significantly improves symptoms compared to placebo.^23^, ^24^, ^25^, ^26^, ^27^, ^28^, ^29^, ^30^, ^31^, ^32^, ^33^ However, no studies have assessed FMT efficacy in adolescents with IBS. This RCT investigated the feasibility and effectiveness of FMT in adolescents aged 16 and 21 years with refractory IBS.

## 2 METHODS

### 2.1 Ethics statement

The study was approved by the Medical Ethics Research Committee of Amsterdam University Medical Centers (AUMC; reference: NL48420.018.16) and conducted in accordance with the Declaration of Helsinki.

### 2.2 Study design and participants

This double‐blind, randomized, placebo‐controlled single‐center pilot study was conducted at AUMC, location Academic Medical Center (AMC), between August 2018 and July 2021. A detailed protocol is published elsewhere.^34^ Eligible participants were adolescents aged 16–21 years with refractory IBS, defined by Rome IV criteria: symptoms persisting ≥ 12 months despite adequate explanation, reassurance, and dietary advice; no response to at least six sessions of psychological treatment; and no response to an adequate dose of at least one pharmacological agent for a minimum of 6 weeks. Additional in‐ and exclusion criteria are listed in Supporting Information S1: Table S1. Healthy fecal donors (>18 years, body mass index 16–25, non‐smokers, regular morning stool pattern) were recruited via posters, staff announcements, emails, and word‐of‐mouth among AMC employees. Donors underwent thorough screening and regular rescreening for infectious diseases, risk factors, and microbiota‐disrupting factors including laboratory testing of feces and blood according to national guidelines.^35^ Matching of donors to patients was based on biological sex and cytomegalovirus (CMV)/Epstein–Barr virus (EBV) serostatus. Further details on donor recruitment and screening are described elsewhere.^36^ All participants provided written informed consent. Patients were randomized (1:1) to receive either two allogeneic FMTs (from healthy donor) or two autologous FMTs (own stool) at baseline and after 6 weeks. Randomization was performed using a computerized random‐number generator in Castor EDC. Both patients and investigators were blinded to allocation. The randomization assistant was the only person aware of treatment allocation and had no further role in the study. During follow‐up, patients could receive other interventions (e.g., cognitive behavioral therapy, pharmacological agents).

### 2.3 Intervention

On the day of transplantation, a nasoduodenal tube was positioned using the Cortrak® device.^37^ Bowel lavage was performed using 1.5–3.5 L of macrogol electrolytes solution (Klean‐Prep®) via the tube. On the day of infusion, both donor (allogeneic) and recipient (autologous) collected a fresh morning stool sample. If no fresh sample was available, the first stool after lavage was used. A research assistant, not involved in the trial, ensured blinding and delivered the randomized, blinded feces to the laboratory where stool was weighed and, depending on consistency, mixed with 200–400 mL saline (0.9% NaCl) under a fume hood. The mixture was filtered through double gauze to remove large debris until 200 mL of fully homogenized suspension was obtained and stored at 4°C. Within 6 h of stool production, the suspension was infused into the duodenum via the nasoduodenal tube.

### 2.4 Patient outcomes

#### 2.4.1 Feasibility outcomes

The primary objective was to assess the study feasibility. Feasibility outcomes included monthly patient recruitment, eligibility, and dropout rates, incidence of serious adverse events related to FMT (predefined as hospitalization or >100 points increase in IBS‐severity scoring system (IBS‐SSS) pain components), and number of provided stool samples. Patient perception and satisfaction with FMT were evaluated using an adapted patient satisfaction and preference questionnaire.^38^

#### 2.4.2 Efficacy outcomes

Efficacy outcomes included treatment success (>50% reduction in abdominal pain intensity and frequency, assessed with the IBS‐SSS) and adequate relief (AR) of IBS symptoms (assessed by a single dichotomous question: “Did you have adequate relief of IBS symptoms over the past week?”). at 6, 12, 24, and 48 weeks post‐FMT.^34^, ^39^ Additional outcomes were the proportion of responders (≥50‐point reduction in IBS‐SSS total score), changes in IBS‐SSS total score, health‐related QoL, anxiety and depression scores, school/work absenteeism, adverse events, and dietary intake. Changes in gut microbiota and metabolome composition were also assessed.

### 2.5 Statistical analysis

The planned sample size was 30 patients; 32 were included due to COVID‐19‐related protocol deviations. In April 2020, two patients received only one FMT due to national COVID‐19 measures; two additional subjects were included to compensate (final *n* = 32).^34^ Analyses were performed on an intention‐to‐treat basis. Missing values were imputed by adding the overall mean change of the variable to the score before the missing value.

Descriptive statistics summarized feasibility outcomes. For efficacy outcomes, independent t‐tests were used for continuous variables with symmetric distribution, and Mann–Whitney *U* tests for skewed data. Categorical variables were compared using *χ* ^2^ or Fisher's exact tests. Due to the pilot nature of the study, no correction for multiple comparisons was applied, including for baseline differences. To analyze within‐patient changes over time, a general linear model (repeated‐measures analysis) was used. Mauchly's Test of Sphericity assessed the assumption of sphericity; if violated, a Greenhouse–Geisser correction was applied. Baseline values were not incorporated in these analyses due to the small sample size. After all statistical analyses, the study was unblinded and participants were informed of their allocation. Statistical significance was set at *α* = 0.05. Analyses were performed using IBM SPSS Statistics for Windows, Version 26.0 (Armonk, NY: IBM Corp).

## 3 RESULTS

**Figure 1:**
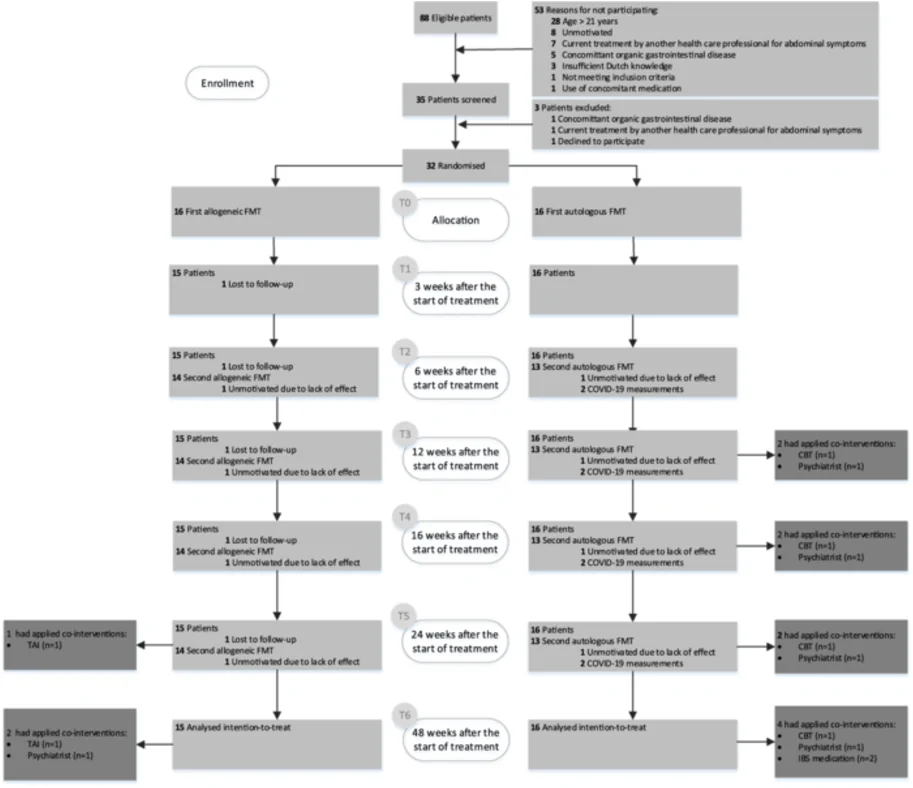
Flowchart. CBT, cognitive behavioral therapy; COVID‐19, Cornoavirus disease 2019; FMT, fecal microbiota transplantation; TAI, transanal irrigation.

A total of 88 patients were interested in participation (Figure 1). After pre‐screening, 53 patients were excluded, and 35 were invited for a screening visit with routine laboratory testing. Three patients were excluded or withdrawn from the study prior to start: abnormal routine laboratory testing: Crohn's disease (*n* = 1), treatment by another health care professional for abdominal symptoms (*n* = 1), and declined positioning of nasoduodenal tube (*n* = 1 Ultimately, 32 patients were randomized to allogeneic (*n* = 16) or autologous (*n* = 16) fecal infusions. No withdrawals occurred due to (serious) adverse events. In the allogeneic group 2/15 (13%) started other interventions after 48 weeks compared to 4/16 (25%) in the autologous group. Details on interventions per group are shown in Figure 1. Dietary intake was evaluated, but only 3/32 patients (9%) completed dietary intake lists, limiting further analysis.

### 3.1 Baseline characteristics

Baseline characteristics of included patients are shown in Table 1. Mean age (standard deviation [SD]) was 18.7 (1.7) years and 23 (72%) were female. Median (interquartile range [IQR]) duration of symptoms was 59 months (33–88). Median (IQR) IBS‐SSS was 335 (250–364). Fifty percent experienced school/work absenteeism, median 8.5 h (IQR 3.0–13.5) in the past week. Clinical features did not differ between groups. Details on stool donors and screenings are listed in Supporting Information S1: Table S2. Eleven donors contributed samples, with eight used for allogenic transplantations. Median donor participation was 14 months (IQR 9–18).

**Table 1:** Baseline characteristics.

Characteristic	Allogeneic	Autologous	*p‐*value
	FMT (*n* = 16)	FMT (*n* = 16)	
Age, mean (SD), years	18.4 (1.6)	18.9 (1.9)	0.385
BMI, kg/m^2^	22.1 (20.3–23.3)	22.5 (19.4–28.4)	0.678
Female, frequency (%)	10 (63%)	13 (81%)	0.433
Male	6 (37%)	3 (19%)	
Type of IBS, frequency (%)			0.731
IBS‐C	5 (31%)	6 (38%)	
IBS‐D	5 (31%)	4 (25%)	
IBS‐M	5 (31%)	6 (48%)	
IBS‐U	1 (6%)	0	
Duration of symptoms, *m*	66 (36–123)	49 (26–84)	0.336
Work/school absenteeism, frequency (%)	7/16 (44%)	10/16 (63%)	0.288
Hours absenteeism in the last week	9 (3–14)	8.5 (3–22.8)	
IBS severity scoring system			
Total scores	325 (250–360)	335 (251–362)	0.955
Severity of abdominal pain	75 (50–80)	68 (53–75)	0.323
Frequency of abdominal pain	80 (50–100)	85 (65–100)	0.580
Severity of abdominal distension	50 (30–70)	48 (36–74)	0.880
Dissatisfaction with bowel habits	70 (40–80)	60 (43–70)	0.184
Interference with quality of life	50 (30–80)	60 (50–78)	0.425
Health‐related quality of life			
Total scores	73.1 (61–86.2)	69.9 (49.6–77.6)	0.318
Dysphoria	70.3 (55.5–87.5)	67.2 (40.6–78.1)	0.250
Interference with activities	58.9 (50.9–83.9)	67.9 (47.3–77.7)	0.925
Body image	87.5 (51.6–100)	62.5 (50–81.3)	0.099
Health worry	83.3 (60.4–91.7)	66.7 (50–75)	0.127
Food avoidance	75 (50–97.9)	64.2 (41.7–75)	0.154
Social reactions	62.5 (45.3–87.5)	71.9 (45.3–81.3)	0.925
Sexual health	93.8 (75–100)	87.5 (75–100)	0.744
Effect on relationships	87.5 (60.4–97.9)	75 (54.2–91.7)	0.370
Generic quality of life			
Physical functioning	90.0 (80.0–98.8)	82.5 (66.3–95.0)	0.102
Role physical	25.0 (0–75.0)	25.0 (0–50.0)	0.798
Role emotional	83.3 (33.3–100.0)	50.0 (33.3–100.0)	0.558
General health	40.0 (26.3–60.8)	38.5 (30.0–58.0)	0.940
Bodily pain	46.5 (31.3–51.0)	51.0 (34.3–58.5)	0.744
Vitality	45.0 (30.0–65.0)	40.0 (31.3–45.0)	0.203
Social functioning	62.5 (25.0–71.9)	50.0 (50.0–75.0)	0.618
Mental health	66.0 (48.0–80.0)	62.0 (49.0–76.0)	0.584
Hospital anxiety and depression scale			
Anxiety score	5.5 (1.0–13.5)	7.0 (4.0–11.0)	0.345
Depression scores	5.0 (1.5–7.0)	6.0 (4.0–9.3)	0.288

*Note*: Data are presented as median (IQR), unless otherwise indicated.

Abbreviations: BMI, body mass index; FMT, fecal microbiota transplantation; IBS, irritable bowel syndrome; IBS‐C, irritable bowel syndrome with constipation; IBS‐D, irritable bowel syndrome with diarrhea; IBS‐M, irritable bowel syndrome mixed type; IBS‐U, irritable bowel syndrome unclassified; IQR, interquartile range; SD, standard deviation.

### 3.2 FMT in adolescents with IBS is feasible and perceived as innovative

Of 35 recruited, 32 (91%) were included for primary endpoint analyses. Two patients (6%) discontinued repetitive FMTs due to lack of effect after the first transplant; two others (6%) due to COVID‐19 measures. No FMT‐related hospitalizations occurred. 1/15 patients reported a significant increase in IBS‐SSS pain score after the first allogeneic FMT, and 2/16 patients reported similar increases after autologous FMTs. All stool samples were provided by 28/31 patients (90%). One patient, unmotivated to provide all morning samples, continued by completing online questionnaires after allogeneic FMTs.

#### 3.2.1 Patient perception and satisfaction

Prior to treatment, FMT was perceived as “innovative” (72%), “unpleasant” (47%), “natural remedy” (25%), and “disgusting” (6%). Patients’ satisfaction (ranging between 0—no discomfort, and 10—severe discomfort) with different steps of FMT procedure are shown in Supporting Information S1: Figure S1. Patients experienced nasoduodenal tube positioning as the most bothersome. Seventy‐five percent would undergo FMTs again if needed.

### 3.3 Allogeneic FMTs significantly reduces IBS symptoms

**Figure 2:**
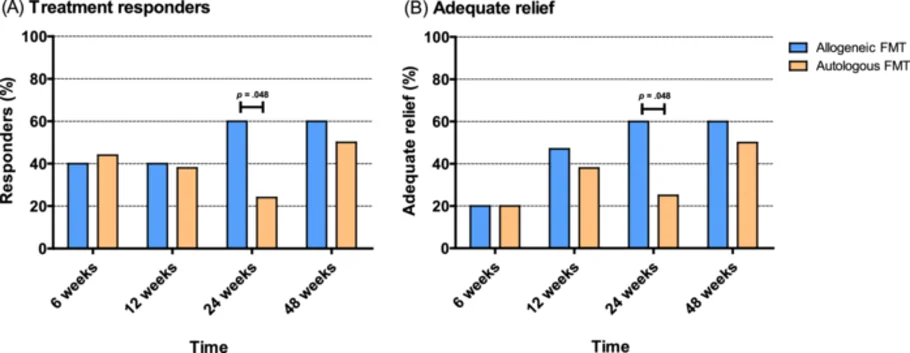
Efficacy of FMT. Time in weeks after first FMT: 6 weeks after start treatment (T2); 12 weeks after start treatment (T3); 24 weeks after start treatment (T5); 48 weeks after start treatment (T6). Treatment responders: patients with ≥50 points reduction in the IBS‐SSS total score. Adequate relief: measured using a single question (“Did you have adequate relief of IBS symptoms [abdominal discomfort/pain, bowel habits, and other symptoms like nausea and bloating] over the past week?”) scored on a dichotomous scale (Yes/No). FMT, fecal microbiota transplantation; IBS‐SSS, irritable bowel syndrome‐severity scoring system.

Treatment success rates did not differ significantly between groups: after 6 weeks (T2), two patients (13%) in the allogeneic group versus one patient (6%) in the autologous group (*p* = 0.600). At 12 weeks (T3), 24 weeks (T5), and 48 weeks (T6) treatment success rates after allogeneic infusion versus autologous infusion were 4/15 (27%) versus 2/16 (13%, *p* = 0.394), 3/15 (20%) versus 2/16 (13%, *p* = 0.654) and 2/15 (13%) versus 2/16 (13%, *p* = 1.000), respectively. Responders and AR rates are shown in Figure 2A,B. Response rates were significantly higher 24 weeks after the first allogeneic FMT (60% [9/15] vs. autologous: 25% [4/16], *χ* ^2^[1] = 3.895, *p* = 0.048). Similarly, after allogeneic FMTs, 60% of patients reported AR at 24 weeks follow‐up compared with 25% of patients after autologous FMTs (*χ* ^2^[1] = 3.895, *p* = 0.048). No significant differences in response or AR rates were found at other time points.

Total IBS‐SSS score decreased significantly over time in both groups (*p* = 0.002, Supporting Information S1: Table S3). At 24 weeks, change from baseline was significantly greater after allogenic FMT (*U* = 70, *z* = –2.001, *p* = 0.045). IBS‐SSS subscores are shown in Supporting Information S1: Table S4. Severity and frequency of abdominal pain significantly improved in both groups (*p* = 0.045 and *p* = 0.002, respectively), whereas abdominal distension, dissatisfaction with bowel habits, and interference with QoL did not. Median subscores between groups did not differ.

**Figure 3:**
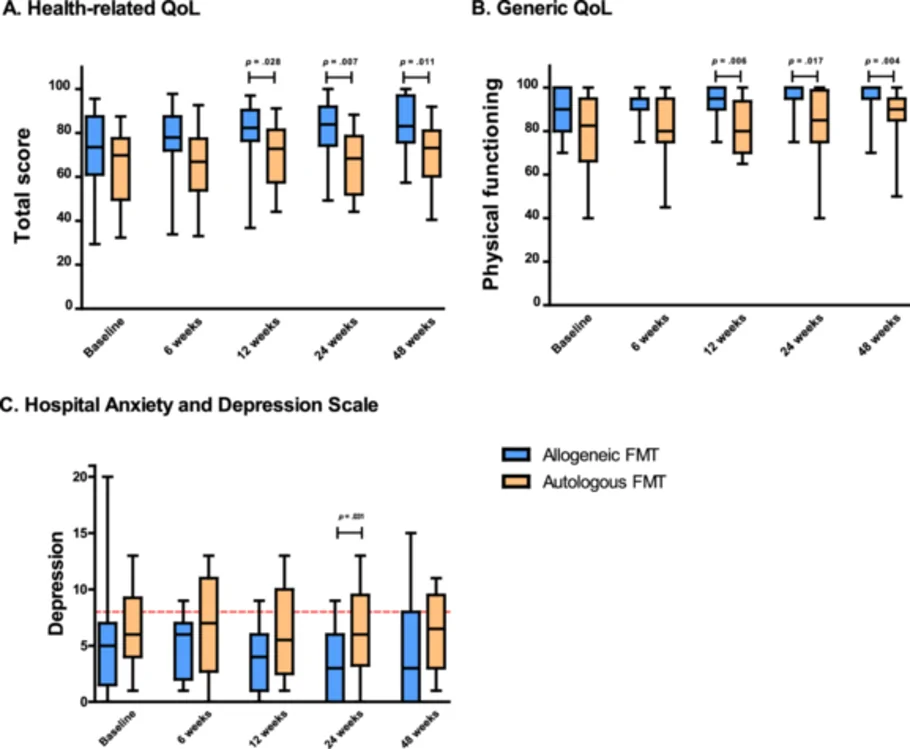
Efficacy outcomes. (A) Health‐related QoL was measured using the IBS‐QoL questionnaire, with scores from 0 (poor) to 100 (excellent); (B) generic QoL was assessed with the SF‐36, covering eight health domains; scores range from 0 (worst) to 100 (best); (C) higher scores indicate higher level of depression (range 0–21). A scale score of ≥8 indicates clinically significant depression. Data are presented as median (IQR); time in weeks after first FMT: 6 weeks after start treatment (T2); 12 weeks after start treatment (T3); 24 weeks after start treatment (T5); 48 weeks after start treatment (T6). FMT, fecal microbiota transplantation; IBS, irritable bowel syndrome; IQR, interquartile range; QoL; quality of life.

Other efficacy outcomes, including school/work absenteeism, health‐related and generic QoL, anxiety, and depression are shown in Supporting Information S1: Table S5. Differences were most significant at 24, all favoring allogeneic FMT. One patient (7%) reported school/work absenteeism 24 weeks after allogeneic FMTs versus 7 (44%) after autologous FMTs (*p* = 0.037). Health‐related QoL (Figure 3A) and physical functioning (Figure 3B) were significantly better after allogeneic FMTs at 12, 24, and 48 weeks, and depression scores were significantly lower at 24 weeks (Figure 3C). Response of the recipients of the allogeneic FMTs appeared to be related to the allocated donors (Supporting Information S1: Figure S2).

After the first FMT 84% (27/32) experienced at least one adverse event, without significant differences between groups (allogeneic 15/16 and autologous 12/16). After the second FMT, 56% (15/27) experienced adverse events (allogeneic 8/14 vs. autologous 7/13). Reported adverse events were mild and transient. Gastro‐intestinal pain/cramp was more common after allogeneic FMT (52% vs. 24%). All adverse events are shown in Supporting Information S1: Table S6.

## 4 DISCUSSION

This pilot study demonstrates that FMT is a feasible and well‐tolerated intervention in adolescents with refractory IBS. The dropout rate was low (<10%), adverse events were mild and transient, and a high proportion of patients (75%) indicated willingness to undergo repeat procedures. All adverse events, including transient diarrhea and nausea, were mild and resolved spontaneously within 3 days. Notably, gastrointestinal pain or cramping occurred significantly more often after allogeneic FMT (52% vs. 24% in the autologous group), which is consistent with findings in adult studies and may reflect transient changes in gut microbial composition rather than effects of bowel lavage.^24^

Patients who received allogeneic FMT reported significantly better health‐related QoL, improved physical functioning, and lower levels of depression compared to those who received autologous FMT. The clinical response to allogeneic FMT increased over time, peaking at 24 weeks and persisting up to 48 weeks. These findings suggest that FMT may have a sustained beneficial effect on symptoms and daily functioning in this difficult‐to‐treat population. The improvement in school/work absenteeism and depression scores further supports the potential broader impact of FMT on psychosocial well‐being and daily life. Although statistically significant, it is important to note that baseline QoL scores were already relatively high in both groups. The absolute magnitude of change was modest, and the boxplots showed overlapping interquartile ranges and considerable individual variability. This suggests that, while some patients experienced meaningful improvement, the overall clinical significance of these changes may be limited. These results are in line with previous RCTs and meta‐analyses in adults with IBS, which demonstrated that allogeneic FMT can lead to significant and sustained improvements in symptom severity, QoL, and psychological well‐being, with effects often peaking several months after treatment and persisting up to 1 year.^24^, ^27^, ^31^ However, as in our study, the absolute differences in these outcomes are modest and should be interpreted in the context of high placebo response rates and the heterogeneity of the IBS population.

In the current study, the clinical response to allogeneic FMTs increased over time, peaking at 24 weeks and lasting up to 48 weeks. Previous FMT trials in adult IBS patients, using single FMT through invasive routes, reported benefits at 12 weeks.^31^, ^32^ While lacking empirical support, one could hypothesize that including a second FMT after 6 weeks into our protocol may have enhanced donor microbiota engraftment, affecting clinical outcomes.^40^ Because FMT was administered both at baseline and again at Week 6, with efficacy assessed from Week 12 onward, it is difficult to disentangle the specific contributions of the first versus the second FMT to the observed clinical response. The delayed peak in response at 24 weeks, with no significant difference at earlier time points, may suggest a cumulative or delayed effect, but this design does not allow us to attribute the timing or durability of the response to a specific infusion.

The study also showed beneficial effects on IBS symptoms with autologous FMTs, though to a lesser extent. Other factors, such as high placebo effect in pediatric IBS and the allowance of other interventions during follow‐up, may have contributed to symptom improvement.^41^ Patients in both groups started co‐interventions (allogeneic: 2/15; autologous: 4/16). Due to the limited sample size, we could not correct for application of other interventions in our analyses. Moreover, not considering baseline characteristics in the efficacy outcome analysis may have enhanced random effects.

Our main challenge was finding safe, eligible, and healthy stool donors, able to provide fresh morning stool samples on the day of transplantation. The obstacles in donor recruitment, screening, follow‐up, and the associated costs have been described previously.^36^ Frozen FMT products could ease logistical challenges of obtaining fresh donor samples. Initially, 18% of eligible donors were excluded due to strict matching criteria based on biological sex and CMV/EBV status. Since no FMT studies had been conducted in adolescent IBS patients, we designed our protocol with extreme caution. Over time, insights have changed as there is no evidence that donor‐recipient compatibility depends on biological sex. Matching on EBV/CMV status is only recommended when FMT is administered to immunosuppressed patients.^42^ For future FMT protocols in pediatric or adolescent IBS patients, the aforementioned donor and recipient matching criteria are therefore not necessary, decreasing the hurdle of finding eligible donors.

Participants found nasoduodenal tube placement the most cumbersome step of the FMT procedure. Alternative routes of transplantation, such as oral FMT capsules, make the intervention less invasive and seems a crucial step to make the intervention acceptable for future standard care. Two studies have assessed the efficacy of FMT capsules in adult IBS patients.^23^, ^25^ In both studies, short duration of the intervention and use of four different fecal donors for production of the FMT capsules may have contributed to the lack of positive results, as donor mix FMT products may have different engraftment effects.^43^ Data on the use of donor FMT capsules in pediatric IBS patients is lacking.

In adult IBS patients, some studies performed FMT by using a mix of healthy donor stool,^25^, ^28^ whereas others used two to four different donors,^23^, ^26^, ^27^ or one single donor for all FMTs.^24^, ^29^ This variability could explain conflicting study results. Our study used feces from eight different donors for all allogeneic FMTs. While our analysis was not adequately powered to detect statistically significant differences between individual donors, we observed trends suggesting that donor‐specific factors may influence treatment response. This highlights the potential importance of donor‐recipient microbiome compatibility.^40^ Moreover, bacterial profile of donors should be stable over time, as stability seems to be associated to effective donors.^24^, ^27^, ^44^

Our pilot study has several limitations. First, the natural course of IBS may have influenced our results. However, we included only patients with symptoms lasting a median of 5 years without response to various treatments, making impact of the IBS natural course likely negligible. Second, the pilot nature of the study meant the sample size was not adequately powered for IBS subtype analysis. Moreover, the inclusion of different IBS subtypes and a wide range of symptom duration and severity may have introduced heterogeneity, limiting generalizability. Lastly, diet is known to modulate microbial composition.^45^ Unfortunately, only 3/32 patients (9%) completed dietary intake lists.

## 5 CONCLUSION

Donor FMT is a promising, feasible, and safe treatment with potentially beneficial effects for adolescents with refractory IBS. The positive findings provide preliminary evidence for development in clinical use. However, it should not yet be offered outside of a research setting. Future studies should include larger sample sizes to replicate these findings and confirm the beneficial effects of FMT in children and adolescents with refractory IBS.

## CONFLICT OF INTEREST STATEMENT

Max Nieuwdorp is in the Scientific Advisory Board of Caelus Pharmaceuticals, the Netherlands and Sensus, the Netherlands. None of these are directly relevant to the current paper. There are no patents, products in development or marketed products to declare. The other authors declare no conflicts of interest.

## Supporting information


**Supplementary Table S1.** Eligibility criteria patients**. Supplementary Table S2.** Demographics, specifications of screening, and reasons of exclusion of donors. **Supplementary Table S3.** IBS‐SSS total score. **Supplementary Table S4.** IBS‐SSS scores for the five items of the scale. **Supplementary Table S5.** Efficacy outcomes. **Supplementary Table S6.** Adverse events. **Supplementary Figure S1.** Patient Perception and Satisfaction. **Supplementary Figure S2.** Tabulation of the response per donor ‐ indicating donor specific effects

## Data Availability

Deidentified individual participant data are available upon request.

## References

[1] Hyams JS , Di Lorenzo C , Saps M , Shulman RJ , Staiano A , van Tilburg M . Functional disorders: children and adolescents. Gastroenterology. 2016;150(6):1456‐1468. 10.1053/j.gastro.2016.02.015 27144632

[2] Rajindrajith S , Devanarayana NM . Subtypes and symptomatology of irritable bowel syndrome in children and adolescents: a school‐based survey using rome III criteria. J Neurogastroenterol Motil. 2012;18(3):298‐304. 22837878 10.5056/jnm.2012.18.3.298 PMC3400818

[3] Mearin F , Lacy BE , Chang L , et al. Bowel disorders. Gastroenterology. 2016;150(6):1393‐1407. 10.1053/j.gastro.2016.02.031 27144627

[4] Sperber AD , Dumitrascu D , Fukudo S , et al. The global prevalence of IBS in adults remains elusive due to the heterogeneity of studies: a Rome Foundation working team literature review. Gut. 2017;66(6):1075‐1082. 26818616 10.1136/gutjnl-2015-311240

[5] Korterink JJ , Diederen K , Benninga MA , Tabbers MM . Epidemiology of pediatric functional abdominal pain disorders: a meta‐ analysis. PLoS One. 2015;10(5):e0126982. 25992621 10.1371/journal.pone.0126982 PMC4439136

[6] Varni JW , Bendo CB , Nurko S , et al. Health‐related quality of life in pediatric patients with functional and organic gastrointestinal diseases. J Pediatr. 2015;166(1):85‐90.e2. 25241177 10.1016/j.jpeds.2014.08.022

[7] Assa A , Ish‐Tov A , Rinawi F , Shamir R . School attendance in children with functional abdominal pain and inflammatory bowel diseases. J Pediatr Gastroenterol Nutr. 2015;61(5):553‐557. 25950089 10.1097/MPG.0000000000000850

[8] Zamani M , Alizadeh‐Tabari S , Zamani V . Systematic review with meta‐analysis: the prevalence of anxiety and depression in patients with irritable bowel syndrome. Aliment Pharmacol Therap. 2019;50(2):132‐143. 31157418 10.1111/apt.15325

[9] Hoekman DR , Rutten J.M.T.M. , Vlieger AM , Benninga MA , Dijkgraaf MGW. Annual costs of care for pediatric irritable bowel syndrome, functional abdominal pain, and functional abdominal pain syndrome. J Pediatr. 2015;167(5):1103‐1108.e2. 26329806 10.1016/j.jpeds.2015.07.058

[10] de Bruijn CM , Rexwinkel R , Gordon M , Sinopoulou V , Benninga MA , Tabbers MM . Dietary interventions for functional abdominal pain disorders in children: a systematic review and meta‐analysis. Expert Rev Gastroenterol Hepatol. 2022;16(4):359‐371. 35311425 10.1080/17474124.2022.2055547

[11] Gordon M , Sinopoulou V , Tabbers M , et al. Psychosocial interventions for the treatment of functional abdominal pain disorders in children: a systematic review and meta‐analysis. JAMA Pediatr. 2022;176(6):560‐568. 35404394 10.1001/jamapediatrics.2022.0313 PMC9002716

[12] Rexwinkel R , Vermeijden NK , Zeevenhooven J , et al. The low FODMAP diet in adolescents functional abdominal in a non‐guided setting: a prospective multicenter cohort study. Eur J Pediatr. 2025;184(2):189. 39934502 10.1007/s00431-025-05999-9 PMC11814023

[13] Rexwinkel R , de Bruijn CMA , Gordon M , Benninga MA , Tabbers MM . Pharmacologic treatment in functional abdominal pain disorders in children: a systematic review. Pediatrics. 2021;147(6):e2020042101. 34045320 10.1542/peds.2020-042101

[14] Rexwinkel R , Vermeijden NK , Zeevenhooven J , et al. Mebeverine and the influence of labeling in adolescents with irritable bowel syndrome or functional abdominal pain not otherwise specified: a 2 × 2 randomized, placebo‐controlled trial. Gastroenterology. 2025;169(1):94‐103. 40074185 10.1053/j.gastro.2025.02.030

[15] Gieteling MJ , Bierma‐Zeinstra SM , Passchier J , Berger MY . Prognosis of chronic or recurrent abdominal pain in children. J Pediatr Gastroenterol Nutr. 2008;47(3):316‐326. 18728528 10.1097/MPG.0b013e31815bc1c1

[16] Drossman DA . Functional gastrointestinal disorders: history, pathophysiology, clinical features and Rome IV. Gastroenterology. 2016;150:1262‐1279.e2. 10.1053/j.gastro.2016.02.032 27144617

[17] Saulnier DM , Riehle K , Mistretta TA , et al. Gastrointestinal microbiome signatures of pediatric patients with irritable bowel syndrome. Gastroenterology. 2011;141(5):1782‐1791. 21741921 10.1053/j.gastro.2011.06.072 PMC3417828

[18] Pittayanon R , Lau JT , Yuan Y , et al. Gut microbiota in patients with irritable bowel Syndrome‐a systematic review. Gastroenterology. 2019;157(1):97‐108. 30940523 10.1053/j.gastro.2019.03.049

[19] Su Q , Tun HM , Liu Q , et al. Gut microbiome signatures reflect different subtypes of irritable bowel syndrome. Gut Microbes. 2023;15(1):2157697. 36573834 10.1080/19490976.2022.2157697 PMC9809927

[20] Rigsbee L , Agans R , Shankar V , et al. Quantitative profiling of gut microbiota of children with diarrhea‐ predominant irritable bowel syndrome. Am J Gastroenterol. 2012;107(11):1740‐1751. 22986438 10.1038/ajg.2012.287

[21] Hollister EB , Oezguen N , Chumpitazi BP , et al. Leveraging human microbiome features to diagnose and stratify children with irritable bowel syndrome. J Mol Diagnost. 2019;21(3):449‐461. 10.1016/j.jmoldx.2019.01.006 PMC6504675 31005411

[22] Halvorson HA , Schlett CD , Riddle MS . Postinfectious irritable bowel syndrome‐‐a meta‐analysis. Am J Gastroenterol. 2006;101(8):1894‐1899; quiz 942. 16928253 10.1111/j.1572-0241.2006.00654.x

[23] Aroniadis OC , Brandt LJ , Oneto C , et al. Faecal microbiota transplantation for diarrhoea‐predominant irritable bowel syndrome: a double‐blind, randomised, placebo‐controlled trial. Lancet Gastroenterol Hepatol. 2019;4(9):675‐685. 31326345 10.1016/S2468-1253(19)30198-0

[24] El‐Salhy M , Hatlebakk JG , Gilja OH , Bråthen Kristoffersen A , Hausken T . Efficacy of faecal microbiota transplantation for patients with irritable bowel syndrome in a randomised, double‐blind, placebo‐controlled study. Gut. 2020;69(5):859‐867. 31852769 10.1136/gutjnl-2019-319630 PMC7229896

[25] Halkjær SI , Christensen AH , Lo BZS , et al. Faecal microbiota transplantation alters gut microbiota in patients with irritable bowel syndrome: results from a randomised, double‐blind placebo‐controlled study. Gut. 2018;67(12):2107‐2115. 29980607 10.1136/gutjnl-2018-316434

[26] Holster S , Lindqvist CM , Repsilber D , et al. The effect of allogenic versus autologous fecal microbiota transfer on symptoms, visceral perception and fecal and mucosal microbiota in irritable bowel syndrome: a randomized controlled study. Clin Transl Gastroenterol. 2019;10(4):e00034. 31009405 10.14309/ctg.0000000000000034 PMC6602784

[27] Holvoet T , Joossens M , Vázquez‐Castellanos JF , et al. Fecal microbiota transplantation reduces symptoms in some patients with irritable bowel syndrome with predominant abdominal bloating: short‐ and long‐term results from a placebo‐controlled randomized trial. Gastroenterology. 2021;160(1):145‐157.e8. 32681922 10.1053/j.gastro.2020.07.013

[28] Johnsen PH , Hilpüsch F , Cavanagh JP , et al. Faecal microbiota transplantation versus placebo for moderate‐to‐severe irritable bowel syndrome: a double‐blind, randomised, placebo‐controlled, parallel‐group, single‐centre trial. Lancet Gastroenterol Hepatol. 2018;3(1):17‐24. 29100842 10.1016/S2468-1253(17)30338-2

[29] Lahtinen P , Jalanka J , Hartikainen A , et al. Randomised clinical trial: faecal microbiota transplantation versus autologous placebo administered via colonoscopy in irritable bowel syndrome. Aliment Pharmacol Therap. 2020;51(12):1321‐1331. 32343000 10.1111/apt.15740

[30] Singh P , Alm EJ , Kelley JM , et al. Effect of antibiotic pretreatment on bacterial engraftment after fecal microbiota transplant (FMT) in IBS‐D. Gut Microbes. 2022;14(1):2020067. 35014601 10.1080/19490976.2021.2020067 PMC8757476

[31] Samuthpongtorn C , Kantagowit P , Pittayanon R , Patcharatrakul T , Gonlachanvit S . Fecal microbiota transplantation in irritable bowel syndrome: a meta‐analysis of randomized controlled trials. Front Med. 2022;9:1039284. 10.3389/fmed.2022.1039284 PMC9669599 36405622

[32] Zhao H. , Zhang X. , Zhang N. , et al. Fecal microbiota transplantation for patients with irritable bowel syndrome: a meta‐analysis of randomized controlled trials. Front Nutr. 2022;9:890357. 35719141 10.3389/fnut.2022.890357 PMC9202577

[33] Halkjær SI , Lo B , Cold F , et al. Fecal microbiota transplantation for the treatment of irritable bowel syndrome: a systematic review and meta‐analysis. World J Gastroenterol. 2023;29(20):3185‐3202. 37346153 10.3748/wjg.v29.i20.3185 PMC10280798

[34] Zeevenhooven J , de Bruijn CMA , Vlieger A , Nieuwdorp M , Benninga MA . Protocol for a pilot randomised, double‐blind, placebo‐controlled trial for assessing the feasibility and efficacy of faecal microbiota transplantation in adolescents with refractory irritable bowel syndrome: FAIS trial. BMJ Paediatr Open. 2020;4(1):e000689. 10.1136/bmjpo-2020-000689 PMC7443263 32864480

[35] Bank NDF . Protocol screening feces donor. [December 2021]. Versie 4. 2016. https://www.ndfb.nl/uploads/7/2/9/7/72970627/160125_screening_protocol_feces_donor_versie_3.pdf

[36] Bénard MV , de Bruijn CMA , Fenneman AC , et al. Challenges and costs of donor screening for fecal microbiota transplantations. PLoS One. 2022;17(10):e0276323. 36264933 10.1371/journal.pone.0276323 PMC9584411

[37] Rao MM , Kallam R , Flindall I , Gatt M , Macfie J . Use of Cortrak‐‐an electromagnetic sensing device in placement of enteral feeding tubes. Proc Nutr Soc. 2008;67(Oce):E109. 18598437 10.1017/S0029665108007416

[38] Kao D , Roach B , Silva M , et al. Effect of oral capsule‐ vs colonoscopy‐delivered fecal microbiota transplantation on recurrent clostridium difficile infection: a randomized clinical trial. JAMA. 2017;318(20):1985‐1993. 29183074 10.1001/jama.2017.17077 PMC5820695

[39] Francis CY , Morris J , Whorwell PJ . The irritable bowel severity scoring system: a simple method of monitoring irritable bowel syndrome and its progress. Aliment Pharmacol Therap. 1997;11(2):395‐402. 9146781 10.1046/j.1365-2036.1997.142318000.x

[40] Schmidt TSB , Li SS , Maistrenko OM , et al. Drivers and determinants of strain dynamics following fecal microbiota transplantation. Nat Med. 2022;28(9):1902‐1912. 36109636 10.1038/s41591-022-01913-0 PMC9499871

[41] Hoekman DR , Zeevenhooven J , van Etten‐Jamaludin FS , et al. The placebo response in pediatric abdominal pain‐related functional gastrointestinal disorders: a systematic review and meta‐analysis. J Pediatr. 2017;182:155‐163.e7. 28081889 10.1016/j.jpeds.2016.12.022

[42] Mullish BH , Quraishi MN , Segal JP , et al. The use of faecal microbiota transplant as treatment for recurrent or refractory *Clostridium difficile* infection and other potential indications: joint British Society of Gastroenterology (BSG) and Healthcare Infection Society (HIS) guidelines. Gut. 2018;67(11):1920‐1941. 30154172 10.1136/gutjnl-2018-316818

[43] Jiang ZD , Jenq RR , Ajami NJ , et al. Safety and preliminary efficacy of orally administered lyophilized fecal microbiota product compared with frozen product given by enema for recurrent *Clostridium difficile* infection: a randomized clinical trial. PLoS One. 2018;13(11):e0205064. 30388112 10.1371/journal.pone.0205064 PMC6214502

[44] El‐Salhy M , Winkel R , Casen C , Hausken T , Gilja OH , Hatlebakk JG . Efficacy of fecal microbiota transplantation for patients with irritable bowel syndrome at 3 years after transplantation. Gastroenterology. 2022;163(4):982‐994.e14. 35709830 10.1053/j.gastro.2022.06.020

[45] Rinninella E , Tohumcu E , Raoul P , et al. The role of diet in shaping human gut microbiota. Best Pract Res Clin Gastroenterol. 2023;62‐63:101828. 10.1016/j.bpg.2023.101828 37094913

