# Supplementary material for: Feasibility and efficacy of fecal microbiota transplantation in adolescents with refractory irritable bowel syndrome: A randomized clinical pilot trial
Source: J Pediatr Gastroenterol Nutr. 2026 Jun 1;83(3):381–9. doi: 10.1002/jpn3.70459 (PMC13539639; doi:10.1002/jpn3.70459)
Supplement: Supplementary file 1 [file JPN3-83-381-s001.docx]

**Supplementary Material**

**Feasibility and efficacy of fecal microbiota transplantation in adolescents with refractory IBS: a randomized clinical pilot trial**

Clara M.A. de Bruijn^1,2,3^, Anna O.J. Oorthuys^1,2,3^, Judith Zeevenhooven^1^, Mark Davids^4^, Johannes H.M. Levels^4^, Arine M. Vlieger^5^, Hilde Herrema^4^, Max Nieuwdorp^4^, Marc A. Benninga^1^

**Affiliations**

1. Emma Children’s Hospital, Amsterdam UMC, University of Amsterdam, Pediatric Gastroenterology, Hepatology and Nutrition, Amsterdam, The Netherlands
2. Amsterdam Reproduction & Development Research Institute, Amsterdam University Medical Centers, Location Academic Medical Center/Emma Children’s Hospital, Amsterdam, the Netherlands
3. Amsterdam UMC, University of Amsterdam, Gastroenterology and Hepatology, Amsterdam Gastroenterology Endocrinology Metabolism Research Institute, Amsterdam, Netherlands.
4. Departments of Internal and Experimental Vascular Medicine, Amsterdam University Medical Centers, Location AMC, Amsterdam, the Netherlands.
5. Department of Pediatrics, St. Antonius Hospital, Nieuwegein, The Netherlands

**Content**

**Supplementary Table S1.**	Eligibility criteria patients

**Supplementary Table S2.**	Demographics, specifications of screening, and reasons of exclusion of donors

**Supplementary Table S3.**	IBS-SSS total score

**Supplementary Table S4.**	IBS-SSS scores for the five items of the scale

**Supplementary Table S5.**	Efficacy outcomes

**Supplementary Table S6.**	Adverse events

**Supplementary Figure S1.**	Patient Perception and Satisfaction.

**Supplementary Figure S2.**	Tabulation of the response per donor - indicating donor specific effects

**Supplementary Table S1.** Eligibility criteria patients

**Inclusion criteria**

Age 16-21 years

Non-smokers

Irritable bowel syndrome (IBS) according to the Rome IV criteria

Refractory symptoms:

1.	Symptoms are present for ≥ 12 months

2.	Patients have received adequate explanation, reassurance and dietary advice for their

symptoms

3.	There is an absence of response to a minimum of six sessions of psychological

treatment, like hypnotherapy or cognitive behavioral therapy

4.	There is an absence of response to an adequate dose of at least one pharmacological

agent used for a minimum of six weeks

Average daily pain rate ≥30mm on the pain component scale of the IBS-SSS^1^

Ability to give informed consent

**Exclusion criteria**

Use of systemic antibiotics in preceding 6 weeks

Use of probiotic treatment in preceding 6 weeks

Use of concomitant medication, including proton pomp inhibitors (PPI) and vasopressine medication
Pain medication in the form of Paracetamol or NSAIDs is allowed

Current use of drugs which influence gastrointestinal motility

(erythromycin, azithromycin, butyl scopolamine, domperidone, peppermint oil capsules, iberogast)

Current treatment by another health care professional for abdominal symptoms

Current treatment by psychologist or psychiatrist for known anxiety or depression disorder

Known swallowing disorder

Known diagnosis of inflammatory bowel disease (i.e. Crohn’s disease or ulcerative colitis)

Known concomitant organic gastrointestinal disease

Known diagnosis of an autoimmune disease

(e.g. hypo- or hyperthyroidism, celiac disease, rheumatoid arthritis)

Condition leading to profound immunosuppression

(HIV, infectious diseases leading to immunosuppression, bone marrow malignancies)

Known diagnosis of cystic fibrosis

Known diagnosis of porphyria

Known pregnancy or current lactation

Use of systematic chemotherapy

Life expectancy < 12 months

Current Intensive Care Unit-stay

XTC, amphetamine or cocaine abuse

Known intra-abdominal fistula

Signs of ileus, diminished passage

Allergy to Macrogol or substituents (e.g. peanuts, shellfish)

History of surgery:

*o   Hemicolectomy (defined as: surgery resulting in a resection of > 0.5 of the colon)*

*o   Presence of a pouch due to surgery*

*o   Presence of stoma*

Insufficient knowledge of the Dutch language

| Donor  *#* | Age  *years* | BMI  *kg/m^2^* | Sex | Donations  *n* | Donations  per allocation ^a^  *n* | Time eligible to donate *months* | Full screenings   *n* | 60 days screenings  *n* | Additional screenings   *n (details)* | Transient positive tests  *n* | Transient pathogens | Reason of exclusion |
| --- | --- | --- | --- | --- | --- | --- | --- | --- | --- | --- | --- | --- |
| 1 | 27 | 21.8 | F | 11 | Allogeneic:6  Autologous:5 | 18 | 5 | 3 | 5 (DFTs) | 2 | *Blastocystis* spp.  (‘rare’ or ‘few’)^b^ | *Blastocystis* spp. (‘moderate’ or ‘many’)^b^ |
| 2 | 28 | 25.3 | F | 11 | Allogeneic:4  Autologous:7 | 15 | 1 | 1 | 0 | 0 | NA | Rehousing |
| 3 | 23 | 23 | M | 10 | Allogeneic:8  Autologous:2 | 9 | 3 | 4 | 1 (SARS-CoV-2) | 0 | NA | NA/end of trial |
| 4 | 25 | 20.2 | M | 6 | Allogeneic:4  Autologous:2 | 14 | 3 | 5 | 0 | 0 | NA | COVID-19 measures |
| 5 | 38 | 24.4 | F | 4 | Allogeneic:-  Autologous:4 | 20 | 2 | 1 | 0 | 0 | NA | NA/end of trial |
| 6 | 25 | 24.0 | F | 4 | Allogeneic: 2  Autologous:2 | 8 | 3 | 5 | 5 (feces viral) | 5 | Noro-, Sapo-, Enterovirus | NA/end of trial |
| 7 | 27 | 19.0 | F | 4 | Allogeneic:-  Autologous:4 | 8 | 4 | 3 | 1 (SARS-CoV-2) | 0 | 0 | No patient match^c^ |
| 8 | 25 | 20.7 | F | 3 | Allogeneic:2  Autologous:1 | 11 | 2 | 1 | 0 | 0 | NA | Long-term travels |
| 9 | 27 | 20.4 | F | 2 | Allogeneic:2  Autologous:- | 14 | 1 | 0 | 1  (serum CBC, CRP) | 0 | NA | *Blastocystis* spp. (‘moderate’ or ‘many’)^b^ |
| 10 | 29 | 23.9 | M | 2 | Allogeneic:-  Autologous:2 | 13 | 2 | 0 | 0 | 0 | NA | *Dientamoeba* *fragilis* + *Blastocystis* spp. |
| 11 | 31 | 21.0 | F | 2 | Allogeneic:2  Autologous:- | 40 | 1 | 0 | 0 | 0 | NA | No patient match^c^ |

**Supplementary Table S2.** Demographics, specifications of screening, and reasons of exclusion of donors

^a^ Since some participants received autologous transplants, not all donor stool samples were utilized. Stool samples from eight donors were utilized to perform all allogeneic transplantations; ^b^ Determined microscopically by an experienced laboratory analyst; ^c^ based on gender or CMV/EBV status.

Abbreviations: CBC, complete blood count; CRP, c-reactive protein; DFT, dual feces test; ESBL, extended spectrum beta-lactamase; NA, not applicable; STEC, shiga toxin-producing Escherichia coli; MDROs, multidrug resistant organisms; SARS-CoV-2, severe acute respiratory syndrome coronavirus 2.

**Supplementary Table S3.** IBS-SSS total score

| Time | Allogeneic  FMT ^a^ | Autologous  FMT ^a^ | *p* -value |
| --- | --- | --- | --- |
| T0 – Baseline | 325 (250 – 360) | 335 (251 – 362) | .955 |
| T1 – 3 weeks after start treatment | 269 (180 – 320) | 273 (212 – 336) | .797 |
| *Change T0 – T1* | *30 (5 – 90)* | *48 (-5 – 98)* | *.968* |
| T2 – 6 weeks after start treatment | 300 (175 – 345) | 282 (241 – 320) | .767 |
| *Change T0 – T2* | *25 (0 – 75)* | *23 (-36 – 89)* | *.812* |
| T3 – 12 weeks after start treatment | 230 (160 – 340) | 250 (236 – 343) | .185 |
| *Change T0 – T3* | *35 (20 – 145)* | *23 (-35 – 100)* | *.313* |
| T4 – 16 weeks after start treatment | 240 (170 – 304) | 290 (214 – 328) | .521 |
| *Change T0 – T4* | *70 (-5 – 140)* | *43 (-45 – 80)* | *.185* |
| T5 – 24 weeks after start treatment | 210 (110 – 285) | 303 (251 – 363) | .053 |
| *Change T0 – T5* | ***75 (5 – 140)*** | ***3 (-54 – 53)*** | ***.045*** |
| T6 – 48 weeks after start treatment | 220 (170 – 295) | 275 (175 – 329) | .353 |
| *Change T0 – T6* | *85 (15 – 160)* | *48 (1 – 106)* | *.353* |

Data are presented as median (IQR); ^a^ Significant decrease over time in both treatment groups

**Supplementary Table S4.** IBS-SSS scores for the five items of the scale

| Time | Abdominal pain  Severity ^a^ | | Abdominal pain  Frequency ^a^ | | Abdominal distension | | Dissatisfaction with  bowel habits | | Interference with  quality of life | |
| --- | --- | --- | --- | --- | --- | --- | --- | --- | --- | --- |
|  | **Allogeneic**  **FMT** | **Autologous**  **FMT** | **Allogeneic**  **FMT** | **Autologous**  **FMT** | **Allogeneic**  **FMT** | **Autologous**  **FMT** | **Allogeneic**  **FMT** | **Autologous**  **FMT** | **Allogeneic**  **FMT** | **Autologous**  **FMT** |
| T0 - Baseline | 75 (50 – 80) | 68 (53 – 75) | 80 (50 – 100) | 85 (65 – 100) | 50 (30 – 70) | 48 (36 – 74) | 70 (40 – 80) | 60 (43 – 70) | 50 (30 – 80) | 60 (50 – 78) |
| T1 - 3 weeks after start treatment | 70 (40 – 85) | 60 (50 – 65) | 60 (40 – 100) | 80 (60 – 100) | 45 (0 – 65) | 50 (0 – 70) | 40 (20 – 75) | 55 (37 – 75) | 55 (30 – 60) | 45 (23 – 70) |
| T2 - 6 weeks after start treatment | 70 (55 – 80) | 61 (56 – 70) | 60 (30 – 100) | 75 (45 – 88) | 50 (30 – 70) | 50 (0 – 70) | 50 (35 – 75) | 58 (36 – 78) | 50 (20 – 70) | 58 (40 – 73) |
| T3 -12 weeks after start treatment | 60 (40 – 75) | 65 (53 – 70) | 40 (20 – 100) | 70 (43 – 80) | 35 ( 0 – 60) | 60 (5 – 74) | 60 (40 – 80) | 50 (35 – 65) | 40 (25 – 60) | 60 (36 – 70) |
| T4 - 16 weeks after start treatment | 60 (45 – 72) | 60 (51 – 74) | 50 (30 – 100) | 75 (50 – 98) | 45 (0 – 59) | 52 (8 – 76) | 50 (30 – 65) | 55 (33 – 73) | 35 (20 – 65) | 48 (40 – 69) |
| T5 - 24 weeks after start treatment | 55 (30 – 70) | 70 (63 – 79) | 40 (10 – 100) | 90 (53 – 100) | 0 (0 – 60) | 55 (33 – 74) | 50 (25 – 75) | 53 (25 – 68) | 35 (5 – 70) | 60 (50 – 70) |
| T6 - 48 weeks after start treatment | 45 (40 – 70) | 58 (50 – 70) | 40 (20 – 100) | 60 (30 – 100) | 40 (0 – 65) | 45 (0 – 70) | 40 (10 – 70) | 60 (30 – 70) | 30 (10 – 65) | 45 (30 – 59) |

Data are presented as median (IQR); ^a^ significant decrease over time in both treatment groups.

**Supplementary Table S5.** Efficacy outcomes

| Characteristic | T0  *Baseline* | | T2  *6 weeks after*  *start treatment* | | T3  *12 weeks after*  *start treatment* | | T5  *24 weeks after*  *start treatment* | | T6  *48 weeks after*  *start treatment* | |
| --- | --- | --- | --- | --- | --- | --- | --- | --- | --- | --- |
|  | **Allogeneic**  **FMT  (n=16)** | **Autologous**  **FMT**  **(n=16)** | **Allogeneic**  **FMT**  **(n=15)** | **Autologous**  **FMT**  **(n=16)** | **Allogeneic**  **FMT**  **(n=15)** | **Autologous**  **FMT**  **(n=16)** | **Allogeneic**  **FMT**  **(n=15)** | **Autologous**  **FMT**  **(n=16)** | **Allogeneic**  **FMT**  **(n=15)** | **Autologous**  **FMT**  **(n=16)** |
| Work/school absenteeism ^a^  *Hours per week* | 7/16 (44%)  *9 (3–14)* | 10/16 (63%) *8.5 (3–22.8)* | 4/15 (27%)  *5.5 (3.5 –12)* | 9/16 (56%)  *9 (5–28)* | 4/15 (27%)  *5.5 (1.5–8)* | 5/16 (31%)  *10 (6.5–34)* | **1/15 (7%)**  *4* | **7/16 (44%)**  *13 (3–18)* | 1/15 (7%)  *23* | 2/16 (13%)  *29 (28–30)* |
| IBS – Quality of Life  Total score ^a^ | 73.1  (61–86.2) | 69.9  (49.6–77.6) | 77.9  (72.1–87.5) | 66.9  (53.7–77.4) | **82.4**  **(76.5–90.4)** | **72.8**  **(57.5–81.4)** | ***83.8***  ***(74.3–91.9)*** | **68.4**  **(52.0–78.5)** | **83.1**  **(75.7–97.1)** | **73.1**  **(60.3–81.1)** |
| IBS – Quality of Life  Dysphoria ^a^ | 70.3  (55.5–87.5) | 67.2  (40.6–78.1) | **78.1**  **(68.8 –87.5)** | **64.1**  **(50.8–80.5)** | 81.3  (71.8–90.6) | 71.9  (56.3–80.5) | **87.5**  **(65.6–96.9)** | **60.9**  **(53.9–82.0)** | **87.5**  **(75–100)** | **70.3**  **(62.5–80.5)** |
| IBS – Quality of Life  Interference with activities ^a^ | 58.9  (50.9–83.9) | 67.9  (47.3–77.7) | 71.4  (60.7–89.3) | 62.5  (47.3–84.8) | 82.1  (75–89.3) | 73.2  (51.8–81.8) | 82.1  (64.3–92.9) | 69.6  (45.5–85.7) | **85.7**  **(75–95.4)** | **73.2**  **(58.0–85.7)** |
| IBS – Quality of Life  Body image ^a^ | 87.5  (51.6–100) | 62.5  (50–81.3) | 87.5  (68.8–100) | 68.1  (57.8–84.4) | **87.5**  **(81.3–93.8)** | **75**  **(61.1–79.7)** | **87.5**  **(75–100)** | **71.9**  **(62.5–85.9)** | **87.5**  **(75–100)** | **68.8**  **(50–81.3)** |
| IBS – Quality of Life  Health worry | 83.3  (60.4 –91.7) | 66.7  (50 –75) | 75  (66.7–91.7) | 66.7  (52.1–75) | 83.3  (75–100) | 75  (54.2–83.3) | **91.7**  **(83.3–91.7)** | **62.5**  **(50–89.6)** | **91.7**  **(75–100)** | **66.7**  **(66.7–81.3)** |
| IBS – Quality of Life  Food avoidance | 75  (50–97.9) | 64.2  (41.7 –75) | 83.3  (75–91.7) | 66.7  (41.7–83.3) | **83.3**  **(66.7–100)** | **62.5**  **(35.4–83.3)** | **91.7**  **(41.7–100)** | **62.5**  **(35.4–83.3)** | **100**  **(58.3–100)** | **58.3**  **(33.3–81.3)** |
| IBS – Quality of Life  Social reactions ^a^ | 62.5  (45.3 –87.5) | 71.9  (45.3 –81.3) | 68.8  (50–81.3) | 62.3  (45.3–75) | 81.3  (56.3–93.8) | 71.9  (57.8–92.2) | 81.3  (68.8–87.5) | 62.5  (45.3–84.4) | 75  (68.8–100) | 78.1  (53.1–92.2) |
| IBS – Quality of Life  Sexual health | 93.8  (75–100) | 87.5  (75 –100) | 100  (87.5–100) | 93.8  (75–100) | 100  (75–100) | 100  (75–100) | 100  (87.5–100) | 100  (75–100) | 100  (87.5–100) | 93.8  (75–100) |
| IBS – Quality of Life  Effect on relationships | 87.5  (60.4–97.9) | 75  (54.2 –91.7) | 83.3  (66.7–100) | 70.8  (52.1–91.7) | **91.7**  **(83.3–100)** | **70.8**  **(71.9–90.6)** | 83.3  (83.3–100) | 75.0  (58.3–91.7) | 91.7  (75–100) | 92.3  (60.4–91.7) |
| Generic – Quality of Life  Physical functioning ^a^ | 90.0  (80.0 – 98.8) | 82.5  (66.3 – 95.0) | 95  (90 – 95) | 80  (75 – 95) | **95**  **(90 – 100)** | **80**  **(70 – 93.8)** | **100**  **(95 – 100)** | **85**  **(75 – 98.8)** | **100**  **(95 – 100)** | **90**  **(85 – 95)** |
| Generic – Quality of Life  Role physical ^a^ | 25.0  (0 – 75.0) | 25.0  (0 – 50.0) | 25  (0 – 75) | 25  (0 – 75) | 25  (0 – 100) | 37.5  (6.3 – 75) | 75  (25 – 100) | 50  (0 – 100) | 50  (0 – 100) | 75  (50 – 100) |
| Generic – Quality of Life  Role emotional | 83.3  (33.3 – 100) | 50.0  (33.3 – 100) | 66.7  (33.3 – 100) | 50.0  (33.3 – 100) | 66.7  (33.3 – 100) | 66.7  (33.3 – 100) | 100  (33.3 – 100) | 83.3  (8.33 – 100) | 100  (33.3 – 100) | 100  (33.3 – 100) |
| Generic – Quality of Life  General health ^a^ | 40.0  (26.3 – 60.8) | 38.5  (30.0 – 58.0) | 45  (25 – 62) | 35  (21.3 – 65.8) | 50  (30 – 72) | 40  (30 – 68.3) | **55**  **(35 – 87)** | **32.5**  **(26.3 – 62)** | 57  (35 – 77) | 37.5  (25.5 – 64.5) |
| Generic – Quality of Life  Bodily pain ^b^ | 46.5  (31.3 – 51.0) | 51.0  (34.3 – 58.5) | 51  (32 – 62) | 41  (25.3 – 51) | 51  (42 – 62) | 46  (34.3 – 51) | **62**  **(42 – 72)** | **51**  **(32 – 51)** | 51  (41 – 72) | 51  (42 – 61.8) |
| Generic – Quality of Life  Vitality | 45.0  (30.0 – 65.0) | 40.0  (31.3 – 45.0) | 55  (35 – 65) | 42.5  (31.3 – 50) | **60**  **(50 – 65)** | **37.5**  **(26.3 – 43.8)** | **60**  **(40 – 75)** | **42.5**  **(31.3 – 48.8)** | 55  (40 – 70) | 40  (31.3 – 50) |
| Generic – Quality of Life  Social functioning | 62.5  (25.0 – 71.9) | 50.0  (50.0 – 75.0) | 62.5  (50 – 75) | 62.5  (37.5 – 71.9) | 75  (50 – 75) | 62.5  (50 – 62.5) | 62.5  (50 – 87.5) | 62.5  (37.5 – 62.5) | 75  (50 – 100) | 62.5  (62.5 – 71.9) |
| Generic – Quality of Life  Mental health | 66.0  (48.0 – 80.0) | 62.0  (49.0 – 76.0) | 72  (64 – 84) | 66  (53 – 84) | 75  (50 – 75) | 62  (50 – 79) | 76  (52 – 84) | 60  (48 – 72) | 72  (56 – 88) | 68  (57 – 80) |
| Anxiety and Depression Scale  Anxiety score | 5.5  (1.0 – 13.5) | 7.0  (4.0 – 11.0) | 4.0  (2.0–8.0) | 7.5  (3.3–10.8) | 3.0  (1.0–10.0) | 6.0  (2.8–10.0) | 3.0  (1.0–6.0) | 7.5  (4.3–10.5) | 5.0  (1.0–11.0) | 8.0  (5.3–10.8) |
| Anxiety and Depression Scale  Depression total score | 5.0  (1.5 – 7.0) | 6.0  (4.0 – 9.3) | 6.0  (2.0–7.0) | 7.0  (2.7–11.0) | 4.0  (1.0–6.0) | 5.5  (2.5–10.0) | **3.0**  **(0–6.0)** | **6.0**  **(3.3–9.5)** | 3.0  (0–8.0) | 6.5  (3.0–9.5) |

Data are presented as median (IQR); ^a^ significant improvement over time in both treatment groups; ^b^ significant improvement over time in both treatment groups, with significant more improvement after allogeneic FMTs; **significant difference** between groups (allogeneic vs. autologous) at specific time point using Mann – Whitney U test, p < .05; **significant difference** between groups (allogeneic vs. autologous) at specific time point using Mann – Whitney U test, p < .01

**Supplementary Table S6.** Adverse events

|  | Allogeneic | | | | Autologous | | | |  |
| --- | --- | --- | --- | --- | --- | --- | --- | --- | --- |
|  | | 1^st^ FMT  (n=16) | 2^nd^ FMT  (n=14) | Total  (n=30) | | 1^st^ FMT  (n=16) | 2^nd^ FMT  (n=13) | Total  (n=29) | *p* -value |
| Gastro-intestinal pain/cramp | | 10 (63) | 6 (43) | **16 (53)** | | 4 (25) | 3 (23) | **7 (24)** | ***.021*** |
| Transient diarrhea | | 6 (38) | 3 (21) | 9 (30) | | 8 (50) | 2 (13) | 10 (34) | *.712* |
| Nausea | | 5 (31) | 3 (21) | 8 (27) | | 3 (19) | 2 (13) | 5 (17) | *.383* |
| Tiredness | | 3 (19) | - | 3 (10) | | 5 (31) | 3 (23) | 8 (28) | *.083* |
| General muscle ache | | 2 (13) | - | 2 (7) | | - | - | 0 (0) | *NA* |
| Abdominal distension | | 1 (6) | - | 1 (3) | | 2 (13) | - | 2 (7) | *.533* |
| Fever | | 1 (6) | - | 1 (3) | | 2 (13) | - | 2 (7) | *.533* |
| Regurgitation | | 1 (6) | - | 1 (3) | | - | - | 0 (0) | *NA* |
| Loss of appetite | | 1 (6) | - | 1 (3) | | 1 (6) | 1 (8) | 2 (7) | *.533* |

Data are presented as n (%) values.

**Supplementary Figure S1.** Patient Perception and Satisfaction.
Data are presented as median (IQR); score ranging from 0 – no discomfort to 10 – severe discomfort. Abbreviations: FMT: fecal microbiota transplantation.

**Supplementary Figure S2.** Tabulation of the response per donor - indicating donor specific effects
Response was defined as ≥50 points reduction in the IBS-SSS total score.
